# Recent Advances in Metal Powder-Based Additive Manufacturing

**DOI:** 10.3390/ma16113975

**Published:** 2023-05-26

**Authors:** Hong Wu, Yaojia Ren, Yingtao Tian, Alberto Orozco Caballero

**Affiliations:** 1 State Key Laboratory of Powder Metallurgy, Central South University, Changsha 410083, China; 2 Department of Engineering, Lancaster University, Lancaster LA1 4YW, UK; 3 Department of Mechanical Engineering, Chemistry and Industrial Design, Universidad Politécnica de Madrid, Ronda de Valencia, 3, 28012 Madrid, Spain

Over the past two decades, laser additive manufacturing technology has evolved rapidly and has been applied in many industrial sectors. However, traditional manufacturing methods such as casting, forging, rolling, and welding are still prevalent around the world [1]. To meet the demand for lightweight and personalized applications in aerospace and biomedical fields, the use of laser powder bed fusion (LPBF) is proliferating and is expected to maintain a high growth rate in the next ten years.

Significant technical advances have been made in the field of metal powder-based additive manufacturing, and a range of high-performance materials have been successfully developed [2,3]. However, the poor printability and unacceptable metallurgical defects are still the primary concerns that limit the adoption of most alloys in the laser-powder bed-fusion process [4]. Therefore, it is essential to re-design the alloy compositions and make them suitable for LPBF which requires a comprehensive understanding of the impact of complex thermal cycles to the microstructure and properties of the materials. In addition, LPBF offers a superior work-hardening effect to the materials, which gives them a chance to overcome the classical strength-ductility trade-off [5]. This also includes the potential effects and consequences of post-treatment on the microstructure and properties. High internal stresses in as-built specimens are inevitable due to the extremely high cooling rates. This may lead to premature material failure when long service periods are required. Furthermore, understanding the hierarchical heterostructure evolution and the corresponding property changes during post-treatment is of great significance for the development of high-strength materials.

The current Special Issue entitled “Recent Advances in Metal Powder-Based Additive Manufacturing” collects the recent research outcomes addressing the main challenges and aiming to provide possible solutions that may revolutionize the metal powder-based additive manufacturing technology and its applications. It is believed that this Special Issue will provide an innovation platform for researchers in this area to communicate and disseminate their most recent ideas and achievements which will facilitate and support scientists and engineers to continuously make contributions to the field.
